# 2-(Penta-1,3-diynyl)-5-(3,4-dihydroxybut-1-ynyl)thiophene, a Novel NQO1 Inducing Agent from *Echinops grijsii* Hance

**DOI:** 10.3390/molecules15085273

**Published:** 2010-08-03

**Authors:** Jing Shi, Xiaoyu Zhang, Hai Jiang

**Affiliations:** 1 Department of Pharmacy, Zhejiang Medical College, No.481 Binwen Rd., Hangzhou 310053, China; E-Mail: shij136@hotmail.com (J.S.); 2 School of Pharmaceutical Sciences, Zhejiang University, Zijingang Campus, No.388 Yuhangtang Rd., Hangzhou 310058, China; E-Mail: zhangxy_zju@hotmail.com (X.Z.); 3 Department of Urology, First Affiliated Hospital, School of Medicine, Zhejiang University, No.79 Qingchun Rd., Hangzhou 310003, China

**Keywords:** *Echinops grijsii* hance, PDDYT, NQO1, Nrf2, glutathione, Akt

## Abstract

A natural alkynol group-substituted thiophene, 2-(penta-1,3-diynyl)-5-(3,4-dihydroxybut-1-ynyl)-thiophene (PDDYT), was isolated from the roots of *Echinops grijsii* Hance. It possessed potent NAD(P)H: quinone oxidoreductase1 (NQO1) inducing activity and could activate Keap1-Nrf2 pathway effectively in murine hepatoma Hepa 1c1c7 cells. Further investigations indicated that the activation of Keap1-Nrf2 pathway by PDDYT might be attributed to the activation of Akt and depleting the cellular glutathione (GSH).

## 1. Introduction

NAD(P)H: quinone oxidoreductase1 (NQO1) is a phase 2 detoxifying enzyme that can convert toxic quinones to hydroquinones and deactivate radicals and electrophiles known to lead to tumorigenesis in normal cellular processes [1,2]. Thus induction of NQO1 is an important detoxification pathway in the life process. The expression of NQO1 is regulated by the Keap1-Nrf2 pathway, in which Keap1 serves as a negative regulator of Nrf2 by proteasomal degradation of Nrf2 through a Keap1-Cul3-Rbx-dependent mechanism [3]. Upon oxidative stress, the Keap1-Nrf2 complex will dissociate, resulting in the transportation of Nrf2 into the nucleus, where it binds to the antioxidant response element (ARE) regions of detoxification genes and accelerates the expressions of phase 2 detoxifying enzymes [4,5,6]. Currently, the most popular screening method for the discovery of NQO1 inducers is a Hepa 1c1c7 cells-based screening platform [7]. A variety of natural inducers of NQO1 have been described, including a number of flavonoids, indoles, isothiocyanates, and dithiolthiones [8].

**Figure 1:**
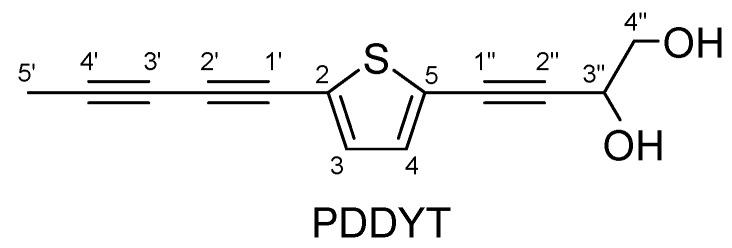
Chemical structure of PDDYT.

2-(Penta-1,3-diynyl)-5-(3,4-dihydroxybut-1-ynyl)-thiophene (PDDYT, Figure 1) is a natural alkynol group-substituted thiophene isolated from the roots of *Echinops grijsii* Hance, a traditional Chinese medicine (TCM) that has a long history of use in China to clear heat, expel miasma and stimulate milk secretion [9,10]. Until now, there are few reports about the biological activity of PDDYT, and only Fokialakis *et al*. have reported that it possessed antifungal activity [11]. In the present study, we demonstrated that PDDYT possessed potent NQO1 inducing activity and could effectively activate the Keap1-Nrf2 pathway. In above process, it was found that the cellular level of glutathione (GSH) decreased markedly after treatment with PDDYT. The compound could also lead to a time-dependent up-regulation of the Ser-473 phosphorylation of Akt. Both the above phenomena might be the mechanisms of action of PDDYT involved in its activation of the Keap1-Nrf2 pathway. We hope our experimental results can provide a more molecular-theoretical basis for the possible application of PDDYT in the future.

## 2. Results and Discussion

### 2.1. PDDYT is a Potent NQO1 Inducing Agent

**Figure 2:**
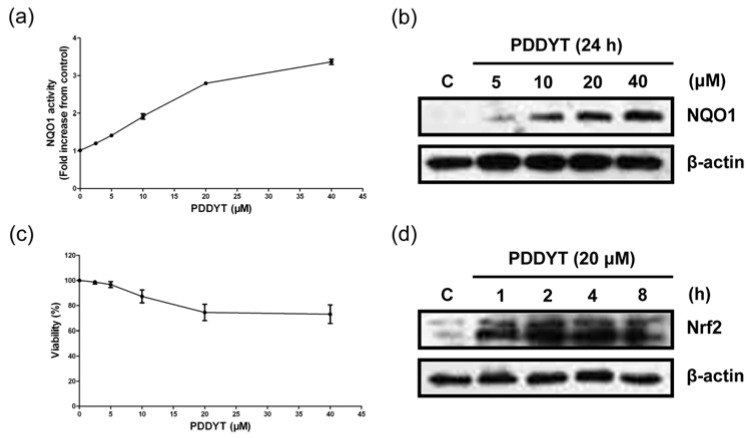
Induction of NQO1 by PDDYT. (a) NQO1 induction assay after Hepa 1c1c7 cells were treated with indicated concentrations of PDDYT for 24 h. (b) Hepa 1c1c7 cells were treated with indicated concentrations of PDDYT for 24 h and analyzed by western blot using anti-NQO1antibody. (c) Crystal violet assay after Hepa 1c1c7 cells were treated with indicated concentrations of PDDYT for 24 h. (d) Nrf2 levels in Hepa 1c1c7 cells were examined after treatment with 20 μM PDDYT for indicated incubation times.

We first investigated the NQO1 inducing activity of PDDYT in Hepa 1c1c7 cells. The results indicated that after the treatment with various concentrations of PDDYT for 24 h, the NQO1 expression increased markedly in a dose-dependent manner and the maximum level was 3.31 ± 0.07-fold of control at 40 μM (Figure 2a and b). 4'-Bromoflavone was tested as the positive control and its NQO1 inducing activity was 4.38 ± 0.12-fold of control at the concentration of 20 μM. The concentration of PDDYT which possessed potent NQO1 inducing activity showed weak cytotoxicity (Figure 2c). It was known that there were two kinds of phase 2 detoxifying enzymes inducers: monofunctional and bifunctional inducers. Monofunctional inducers’ phase 2 detoxifying enzymes inducing activities were via activating Keap1-Nrf2 pathway [5], while bifunctional inducers’ attributed to the activation of the aryl hydrocarbon (Ah) receptor-xenobiotic response element (XRE) pathway [12]. We then investigated the alternation of Nrf2 expression involved in the NQO1 inducing activity of PDDYT. Time course study indicated that after treatment with 20 μM PDDYT for 1 h, Nrf2 levels in Hepa 1c1c7 cells increased obviously (Figure 2d). The results suggested that PDDYT could activate the Keap1-Nrf2 pathway and thus lead to the induction of NQO1.

### 2.2. The Activation of Keap1-Nrf2 Pathway of PDDYT is via Activating Akt and Depleting Cellular GSH

**Figure 3:**
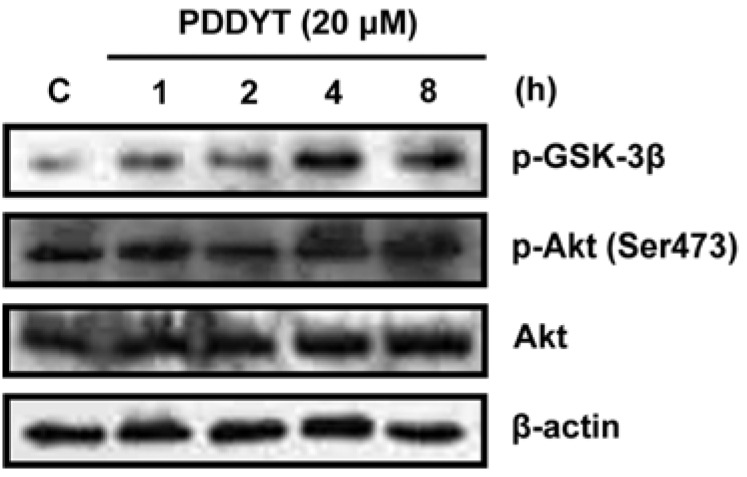
The p-GSK-3*β*, p-Akt (Ser 473) and Akt expressions after Hepa 1c1c7 cells were treated with 20 μM PDDYT for indicated incubation times.

It is known that the activation of Akt pathway could result in the activation of Nrf2 [13,14], so we next investigated whether the activation of Nrf2 by PDDYT can be attributed to the activation of Akt. As shown in Figure 3, treatment of Hepa 1c1c7 cells with 20 μM PDDYT resulted in a time-dependent up-regulation of the Ser-473 phosphorylation of Akt, but no significant change in total Akt protein levels.

**Figure 4:**
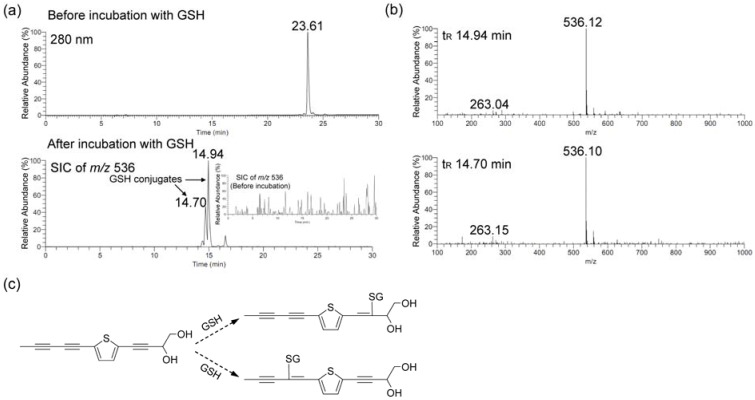
LC-MS analysis of the conjugation between PDDYT and GSH. (a) HPLC chromatogram before and SIC chromatogram after incubation PDDYT (400 μM) with GSH (2 mM) for 2 h. (b) The corresponding mass spectra of PDDYT-GSH conjugates. (c) The proposed reaction between PDDYT and GSH.

An Akt downstream protein, GSK-3*β*, whose phosphorylation is increased in response to Akt activation and thus could be considered as an indication of Akt activity was also up-regulated after the PDDYT treatment (Figure 3). Therefore, the activation of the Keap1-Nrf2 pathway by PDDYT might be attributed to the activation of Akt pathwayIn previous study, it was demonstrated that depleting the cellular level of GSH by electrophilic agents associated with the activation of the Keap1-Nrf2 pathway and induction of phase 2 detoxifying enzymes [15]. Since the chemical structure of PDDYT contains an electron-deficient center and might undergo Michael addition reactions with GSH, we then investigated whether PDDYT could influence the cellular level of GSH. First, PDDYT (400 μM) was incubated with GSH (2 mM) *in vitro* and then the incubation solution was analyzed by LC-MS. The result indicated that PDDYT could conjugate with GSH effectively (Figure 4a). We noticed that there were two peaks in the selected ion current (SIC) chromatogram after incubation of PDDYT with GSH (with the retention times of 14.94 and 14.70 min), the corresponding mass spectra of which were almost the same (Figure 4b). Since position C-2' and position C-2'' of PDDYT were both electron-deficient centers and thus could be attacked by GSH, the two peaks were both identified as PDDYT-GSH conjugates. The proposed reaction between PDDYT and GSH was shown in Figure 4c.

**Figure 5:**
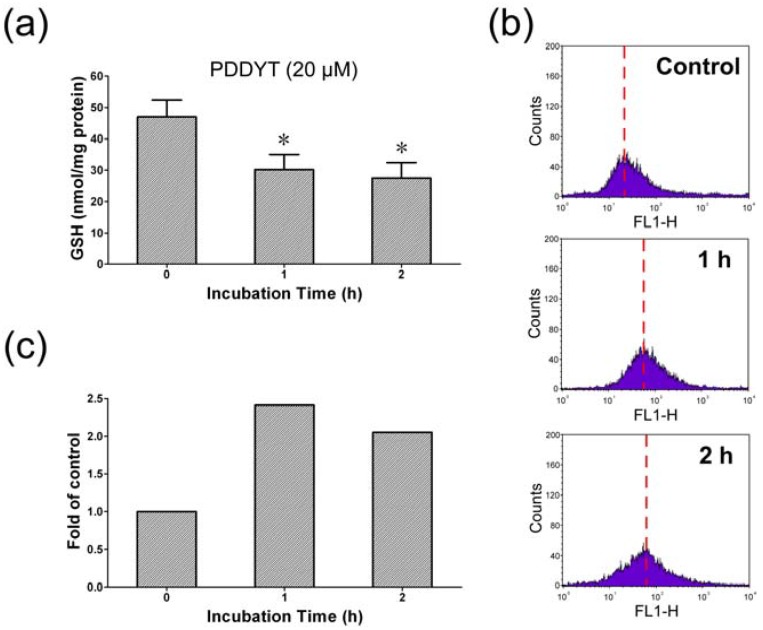
PDDYT mediated the depletion of the cellular GSH. (a) Cellular GSH levels after Hepa 1c1c7 cells were exposed to 20 μM PDDYT for 1 and 2 h. *, treatment groups were significantly different from the control group (P < 0.05) using Student’s *t* test, with n = 3. (b) The ROS generation after Hepa 1c1c7 cells treated with 20 μM PDDYT for 1 or 2 h. (c) Quantification of ROS generation after treatment with PDDYT for 1 and 2 h.

As shown in Figure 5a, the concentration of GSH in Hepa 1c1c7 cells in control experiment (time 0) was 46.98 ± 5.44 nmol/mg protein. When Hepa 1c1c7 cells were treated with 20 μM PDDYT for 1 or 2 h, the GSH level decreased to 30.18 ± 4.85 and 27.43 ± 4.99 nmol/mg protein respectively. Because the decrease in intracellular GSH will lead to the production of ROS [16], we then investigated the change in ROS production in PDDYT-treated Hepa 1c1c7 cells. As shown in Figure 5b, an increase in ROS production was obvious after 1 and 2 h exposure of 20 μM PDDYT. The results suggested that PDDYT could conjugate with and potently decrease the cellular GSH levels, which might be another mechanism involved in its Nrf2 and NQO1 inducing activities.

## 3. Conclusions

In this paper, a natural alkynol group-substituted thiophene PDDYT was isolated from *E. grijisii*. It showed potent NQO1 inducing activity and could effectively activate the Keap1-Nrf2 pathway in Hepa 1c1c7 cells. Further investigations indicated that the activation of the Keap1-Nrf2 pathway by PDDYT might be attributed to an activation of the Akt pathway and depletion of the cellular levels of GSH. Based on the results of the study, PDDYT certainly merits further comparative pharmacological study for the future.

## 4. Experimental

### 4.1. Materials and Reagents

3-(4,5-Dimethylthiazol-2-yl)-2,5-diphenyltetrazolium bromide (MTT) and GSH were purchased from Sigma (St. Louis, MO, USA). 5-(and-6)-carboxy-2',7'-dichlorofluorescin diacetate (carboxy-DCFDA) was purchased from Invitrogen (Carlsbad, CA, USA). All the antibodies were purchased from Santa Cruz Biotechnology (Santa Cruz, CA, USA). The Total Glutathione Assay Kit was purchased from Beyotime. HPLC-grade acetonitrile (Merck, Darmstadt, Germany) and formic acid (Tedia, Fairfield, OH, USA) were utilized for the HPLC analysis. Deionized water was prepared using a Milli-Q system (Millipore, Bedford, MA, USA).

### 4.2. Isolation of PDDYT from E. grijisii

Roots of *E. grijsii* were collected in Bozhou, Anhui Province, People’s Republic of China, in June 2006. The plant material was identified by the authors and a voucher specimen (No. EGH060703) was deposited in the herbarium of the School of Pharmaceutical Sciences, Zhejiang University. The air-dried roots (15 kg) were extracted with 95% ethanol at 95 °C (1 L × 3) to give a crude ethanol extract. The residue was dissolved in H_2_O (1 L) and then extracted with petroleum ether (1 L × 3) and dichloromethane (1 L × 3). The dichloromethane fraction (140 g) was subjected to silica gel column chromatography (200-300 mesh, 12 × 120 cm) and eluted with a petroleum ether-acetic ether gradient system [1:0, 200:1, 100:1, 50:1, 30:1, 20:1, 10:1, 5:1, 1:1, 0:1 (v/v)] to give 10 fractions (Fr. 1-10). Fr. 8 was further separated by preparative HPLC using acetonitrile/H_2_O as the mobile phase (40% acetonitrile maintained for 10 min and increased to 70% in 20 min, flow rate 10 mL/min) to yield PDDYT (110.6 mg, t_R_ 21.4 min). The preparative HPLC experiments were performed using an Agilent 1200 HPLC system (Agilent Technologies, Waldbronn, Germany) coupled with a Zorbax SB-C_18_ column (21.2 mm × 250 mm, 10 μm). The ^1^H- (500 MHz, CDCl_3_) and ^13^C-NMR (125 MHz, CDCl_3_) data and the HSQC and HMBC spectra of PDDYT are shown in Table 1S, Figure 2S and Figure 3S (Supplementary Material). The ^1^H-NMR spectrum showed resonances for two aromatic proton signals at δ 7.14 (d, *J* = 4.0 Hz, H-3) and 7.07 (d, *J* = 4.0 Hz, H-4), indicating the presence of one thiophene ring. It also presented signals for a methyl proton at δ 2.02 (s, H-5'), two oxygenated methylene protons at δ 3.67 (m, H-4'') and an oxygenated methenyl proton at δ 4.55 (t, *J* = 6.0 Hz, H-3''). The ^13^C-NMR spectrum indicated six alkyne carbons at δ 78.9 (C-1'), δ 63.3 (C-2'), δ 65.5 (C-3'), δ 83.2 (C-4'), δ 76.9 (C-1'') and δ 93.2 (C-2''). In the HMBC spectrum, long-range correlations were observed from H-3 to C-3', from H-4 to C-1'' and C-2'', from H-3'' to C-1'', C-2'', C-4'' and C-5, and from H-5' to C-1' and C-2' (Figure 1S, Supplementary Material), indicating that a penta-1,3-diynyl group and a 3,4-dihydroxybut-1-ynyl group were attached to the thiophene ring at C-2 and C-5 respectively. Therefore, the structure of the compound was determined as 2-(penta-1,3-diynyl)-5-(3,4-dihydroxybut-1-ynyl)-thiophene. The purity of PDDYT was greater than 98% according to HPLC analysis (Figure 4S, Supplementary Material) based on a peak area normalization method.

### 4.3. Cell Culture

Hepa 1c1c7 murine hepatoma cells (purchase from ATCC) were maintained in α-minimum essential medium supplemented with 0.1% penicillin-streptomycin and 10% fetal bovine serum. The cells were cultured in 5% CO_2_ at 37 °C.

### 4.4. Crystal Violet Assay for Determining Cell Viability

Hepa lclc7 cells were seeded in 96-well plates at a density of 1 × 10^4^ cells/well. After 24 h, different concentrations of PDDYT were added into each well for an additional 24 h. Then after decanting the media, 0.2% crystal violet in 2% ethanol solution (150 μL) was added into each well and incubated for 10 min. The plates were rinsed three times with water. The bound dye was solubilized in 0.5% SDS in 50% ethanol solution (200 μL). The absorbance of each well was scanned at 550 nm. Experiments were carried out three times on separate occasions.

### 4.5. NQO1 Induction Assay

Hepa lclc7 cells were seeded in 96 well plates at a density of 1 × 10^4^ cells/well. After 24 h, different concentrations of PDDYT were added into each well for an additional 24 h. Then the media was decanted, 0.8% digitonin and 2 mM EDTA solution (pH 7.8) (50 μL) were added into each well and incubated for 10 min. After incubation, a mixed solution containing MTT (0.72 mM), bovine serum albumin (0.67 mg/mL), 0.5 M Tris-HCl, 1.5% Tween 20, 5 μM FAD, 150 mM glucose-6-phosphate, 2 units/mL glucose-6-phosphate dehydrogenase, 50 mM NADP and 50 mM menadione (200 μL) was added into each well. After incubation for 5 min, the absorbance of each well was scanned at 550 nm. The specific activity of NQO1 was determined by measuring NADPH-dependent menadione-mediated reduction of MTT to blue formazan. Experiments were carried out three times on separate occasions.

### 4.6. Western Blot Analysis

After treatment with PDDYT, Hepa 1c1c7 cells were lysed in 150 mM NaCl, 50 mM Tris-HCl (pH 7.4), 1% Triton X-100 containing 1 mM phenylmethylsulphonyl fluoride (PMSF) at 4 °C for 30 min and then centrifuged at 12,000 rpm for 15 min to obtain the total protein. Sixty μg of protein from total protein lysates were separated by sodium dodecyl sulfatepolyacrylamide gel electrophoresis (SDS-PAGE) on a 12% polyacrylamide gel. Proteins were transferred onto nitrocellulose membranes (Bio-Rad, Hercules, CA), probed with primary antibodies and horseradish peroxidase (HRP) conjugated secondary antibodies and then visualized using enhanced chemiluminescence (ECL) western blotting detection reagents (Millipore, Billerica, MA, USA).

### 4.7. Glutathione (GSH) Assay

The total GSH levels were measured by the enzymatic recycling method using glutathione reductase and 5',5'-dithio-bis(2-nitrobenzoic acid) (DTNB). GSH is oxidized by DTNB and reduced by NADPH in the presence of glutathione reductase. The formation of 2-nitro-5-thiobenzoic acid (TNB) is monitored at 412 nm. GSSG was determined after removing of GSH with 2-vinylpyridine. The levels of GSH were calculated from the difference between concentrations of total GSH and GSSG. The intracellular levels of GSH were calculated based on cellular protein concentration.

### 4.8. Detection of Intracellular ROS

After treatment with PDDYT, carboxy-DCFDA was added into the culture medium at a final concentration of 15.0 μM and incubated at 37 °C for an additional 30 min. Then cells were harvested, washed three times with PBS and measured by FACSCalibur using an argon laser at 488 nm and a 525 nm bandpass filter.

### 4.9. Liquid Chromatography-Tandem Mass Spectrometry (LC-MS) Analysis of GSH Conjugates

PDDYT (400 μM) was incubated with GSH (2 mM) in a total volume of 200 µL of 50 mM Tris-HCl buffer solution (pH 8.0) at 37 °C for 2 h. The reaction solution was analyzed using Thermo Finnigan LCQ Deca XP^plus^ ESI ion trap mass spectrometer (San Jose, CA, USA) equipped with an Agilent 1100 HPLC system (Agilent Technologies, Waldbronn, Germany) and Zorbax SB-C_18_ column (4.6 mm × 250 mm, 5 μm, Agilent Technologies, USA). The mobile phase consisted of HCOOH/H_2_O = 0.2:100 (A ) and HCOOH/CH_3_CN = 0.2:100 (B ). A 30 min gradient was used from 10% B to 100% B. The flow rate was 0.5 mL/min and the column temperature was set at 30 °C. The ultraviolet (UV) spectrum was recorded at 280 nm. The MS operating parameters were as follows: collision gas, ultra-high-purity helium (He); nebulizing gas, high-purity nitrogen (N_2_); ion spray voltage, -4.5 kV; sheath gas (N_2_), 5 arbitrary units; capillary temperature, 275 °C; capillary voltage, -15 V; tube lens offset voltage, -30 V. The collision energy for collision-induced dissociation (CID) was between 30% and 45%, and the isolation width of precursor ions was 3.0 Th.

## Supplementary Materials

Supplementary File 1
