# Supplementary material for: 2-(Penta-1,3-diynyl)-5-(3,4-dihydroxybut-1-ynyl)thiophene, a Novel NQO1 Inducing Agent from Echinops grijsii Hance
Source: Molecules. 2010 Aug 3;15(8):5273–81. doi: 10.3390/molecules15085273 (PMC6257798; doi:10.3390/molecules15085273)

Table 1S. ^{1}H and ^{13}C NMR data of PDDYT (in CD_3OD).

Position	δ_H	δ_C
2		123.3
3	7.14 (d, *J* = 4.0 Hz)	133.6
4	7.07 (d, *J* = 4.0 Hz)	131.9
5		124.6
1'		78.9
2'		63.3
3'		65.5
4'		83.2
5'	2.02 (s)	2.9
1"		76.9
2"		93.2
3"	4.55 (t, *J* = 6.0 Hz)	63.3
4"	3.67 (m)	65.6

Figure 1S. The key HMBC correlations of PDDYT.

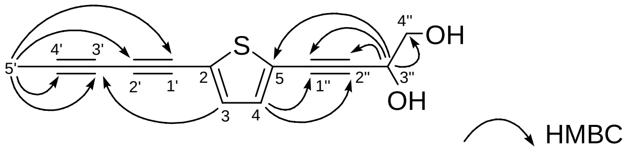

Figure 2S. The ^{1}H NMR (500 MHz, $CDCl_3$) and ^{13}C NMR (125 MHz, $CDCl_3$) spectra of PDDYT.

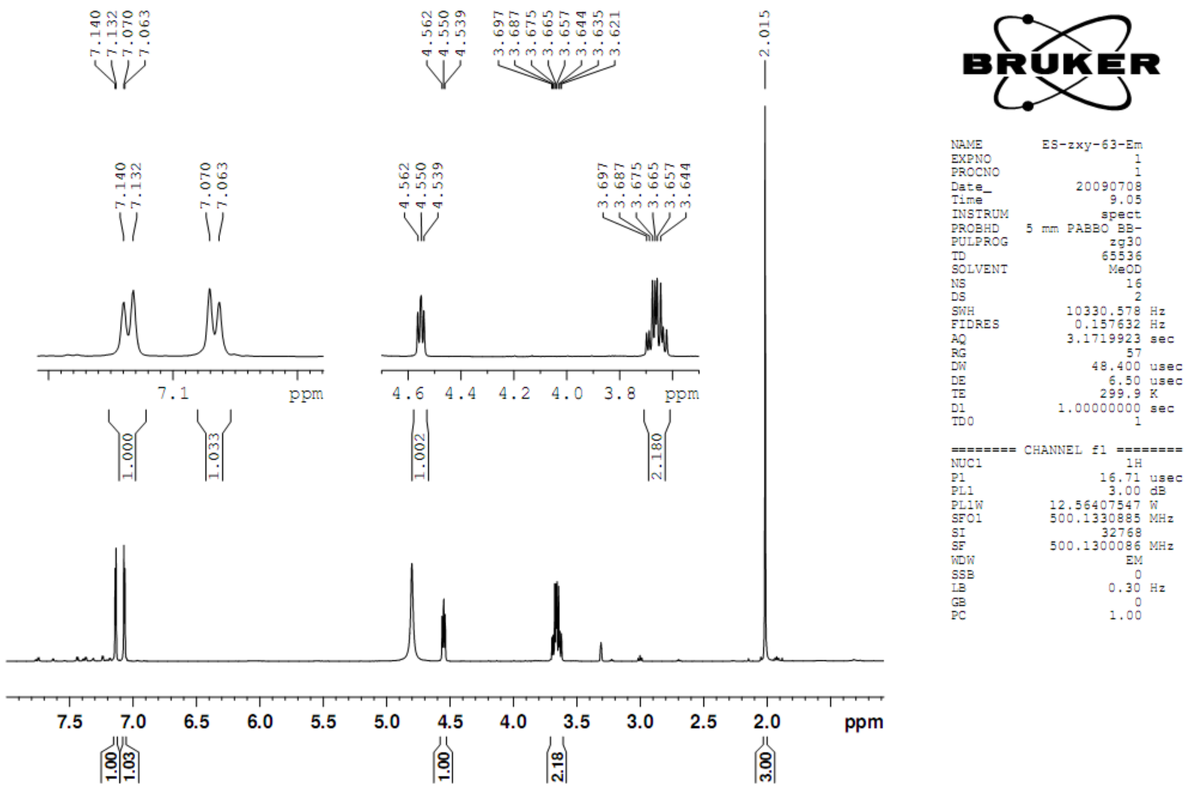

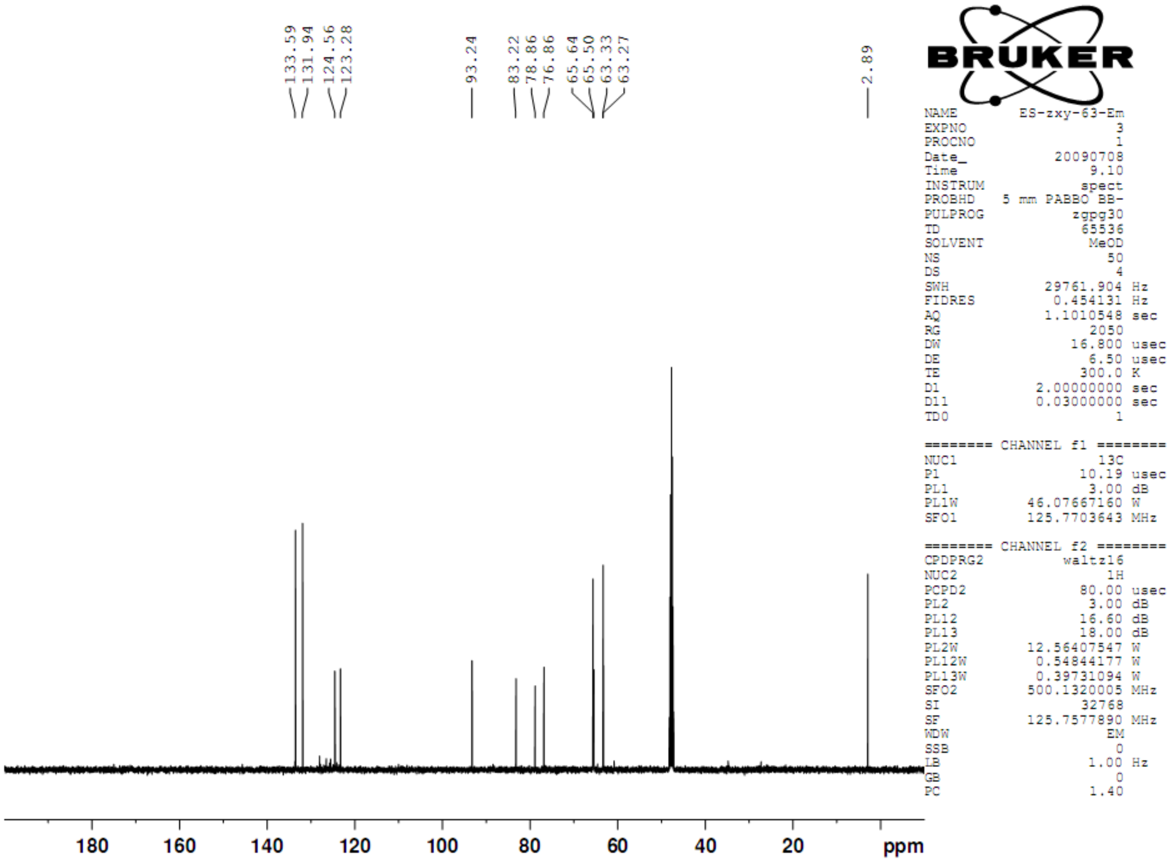

Figure 3S. The HSQC and HMBC spectra of PDDYT.

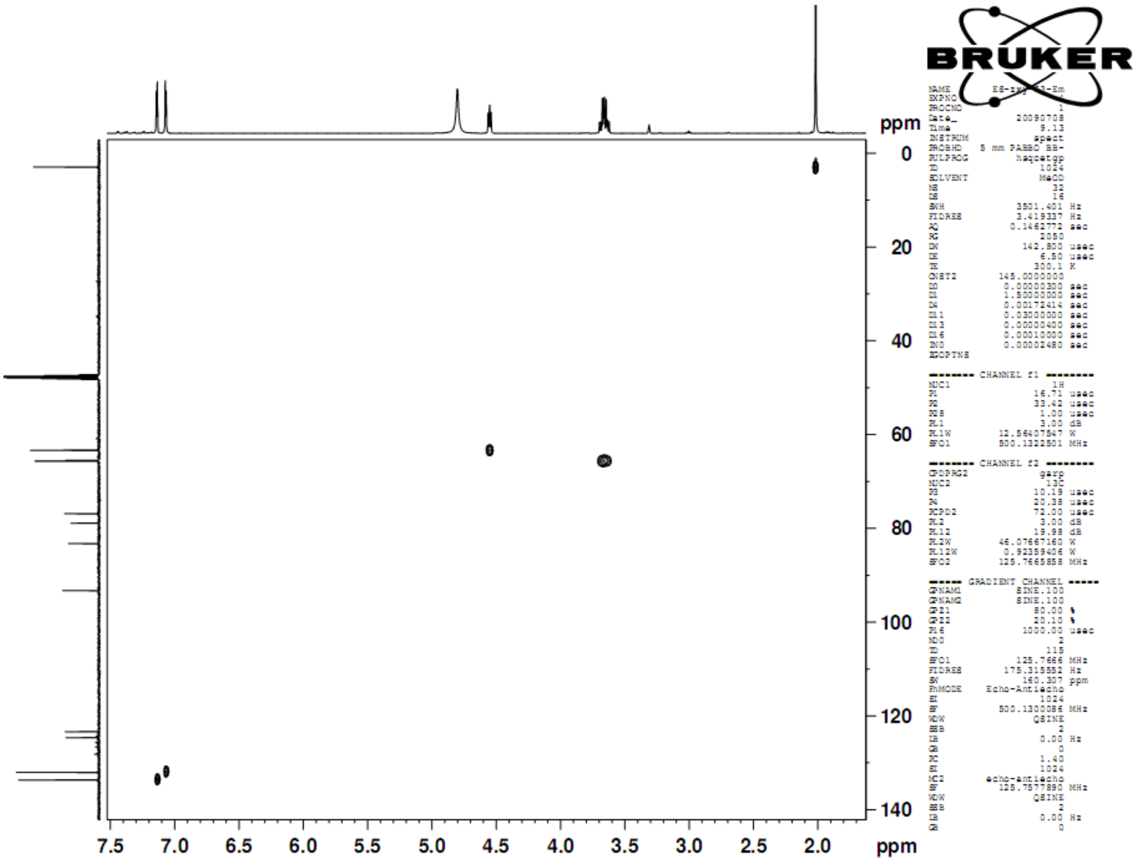

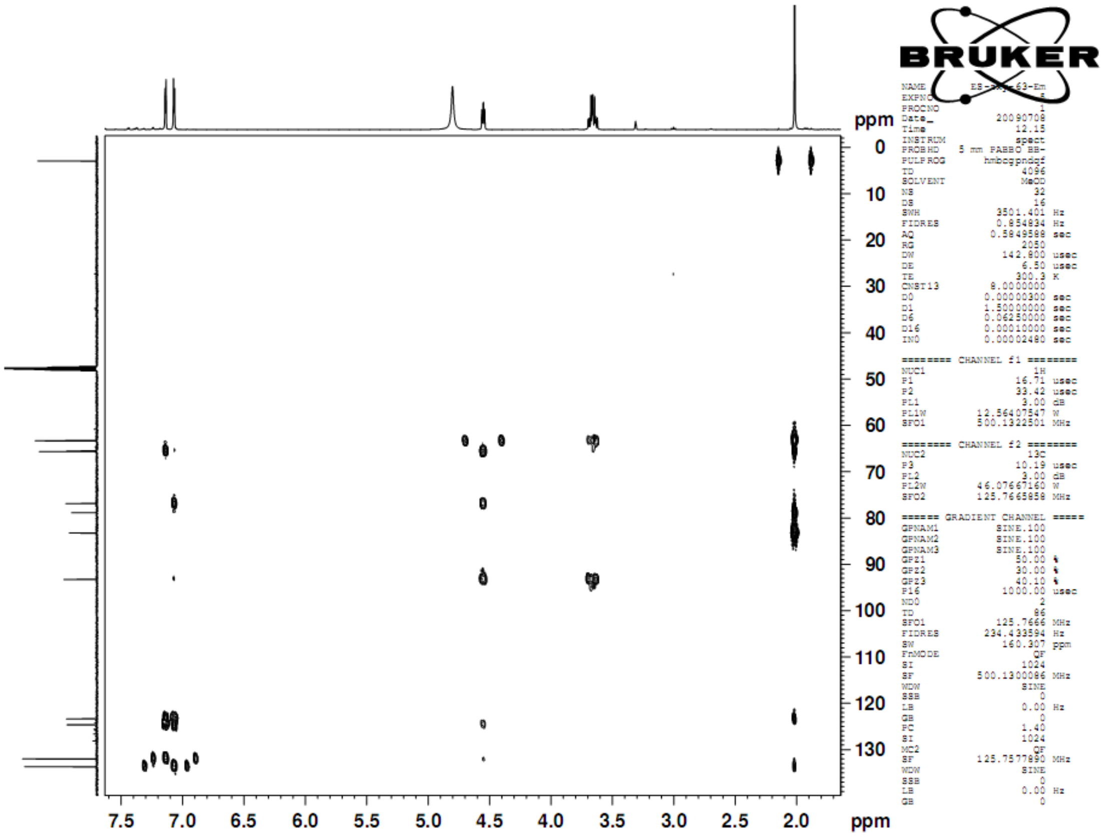

Figure 4S. HPLC analysis of the purity of PDDYT. UV λ = 280 nm.

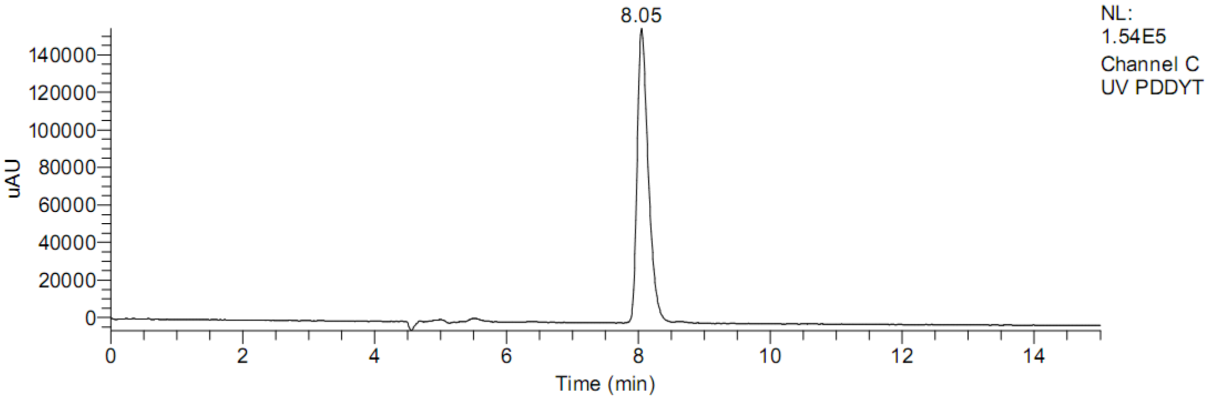

Supplement: Supplementary File 1 [file molecules-15-05273-s001.pdf]
